# Establishing chronic condition concordance and discordance with diabetes: a Delphi study

**DOI:** 10.1186/s12875-015-0253-6

**Published:** 2015-03-28

**Authors:** Elizabeth M Magnan, Rebecca Gittelson, Christie M Bartels, Heather M Johnson, Nancy Pandhi, Elizabeth A Jacobs, Maureen A Smith

**Affiliations:** Department of Family and Community Medicine, University of California, Davis, UC Davis School of Medicine, 4860 Y street, suite 2320, Sacramento, CA 95817 USA; Health Innovation Program, University of Wisconsin School of Medicine and Public Health, 800 University Bay Drive Suite 210, Madison, WI 53705 USA; Department of Medicine, University of Wisconsin, Madison, WI USA; Department of Family Medicine, University of Wisconsin, Madison, WI USA; Department of Population Health Sciences, University of Wisconsin, Madison, WI USA; Department of Surgery, University of Wisconsin, Madison, WI USA

**Keywords:** Delphi, Diabetes, Concordance, Discordance, Multimorbidity, Multiple chronic conditions

## Abstract

**Background:**

The vast majority of patients with diabetes have multiple chronic conditions, increasing complexity of care; however, clinical practice guidelines, interventions, and public reporting metrics do not adequately address the interaction of these multiple conditions. To advance the understanding of diabetes clinical care in the context of multiple chronic conditions, we must understand how care overlaps, or doesn’t, between diabetes and its co-occurring conditions. This study aimed to determine which chronic conditions are concordant (share care goals with diabetes) and discordant (do not share care goals) with diabetes care, according to primary care provider expert opinion.

**Methods:**

Using the Delphi technique, we administered an iterative, two-round survey to 16 practicing primary care providers in an academic practice in the Midwestern USA. The expert panel determined which specific diabetes care goals were also care goals for other chronic conditions (concordant) and which were not (discordant). Our diabetes care goals were those commonly used in quality reporting, and the conditions were 62 ambulatory-relevant condition categories.

**Results:**

Sixteen experts participated and all completed both rounds. Consensus was reached on the first round for 94% of the items. After the second round, 12 conditions were concordant with diabetes care and 50 were discordant. Of the concordant conditions, 6 overlapped in care for 4 of 5 diabetes care goals and 6 overlapped for 3 of 5 diabetes care goals. Thirty-one discordant conditions did not overlap with any of the diabetes care goals, and 19 overlapped with only 1 or 2 goals.

**Conclusions:**

This study significantly adds to the number of conditions for which we have information on concordance and discordance for diabetes care. The results can be used for future studies to assess the impact of concordant and discordant conditions on diabetes care, and may prove useful in developing multimorbidity guidelines and interventions.

**Electronic supplementary material:**

The online version of this article (doi:10.1186/s12875-015-0253-6) contains supplementary material, which is available to authorized users.

## Background

Most adults with diabetes have at least one other chronic condition, and almost half have 5 or more other conditions [1-3]. Multimorbiditiy increases with age, but the majority of persons with multimorbidity are middle aged [4]. Patients with more chronic conditions has been shown in some studies to have better or similar care as those with fewer conditions, partially through increased interactions with the health care system [3,5]. Other studies, however, have suggested that the presence of more conditions are associated with increased mortality [6]. The differing impact of multiple chronic conditions on diabetes care could be due to differences in specific comorbidities’ interactions with diabetes care [1-3].

Currently, we are limited in our knowledge of which comorbidities may improve or inhibit optimal diabetes care [7,8]. We need to understand the interaction between chronic conditions in order to provide adequate care for patients with diabetes and multimorbidity [8]. Current diabetes care guidelines directly address the co-management of diabetes and only a limited number of comorbidities, and remain silent or provide broad, nonspecific guidance for other co-occurring conditions [9,10]. When caring for multimorbid patients, the application of single-condition guidelines may lead to the provision of contradictory and potentially harmful care [7]. Additionally, public reporting metrics are derived from clinical care guidelines, and the presence of public reporting metrics can shape clinical care, so it is particularly valuable to understand the influence of multiple chronic conditions on diabetes care in order to have meaningful quality reports [11,12]. Public reporting could be more meaningful if personalized to individual patients’ comorbidities [13]. Further, effective interventions in multiple chronic conditions are limited, with few evidence-based interventions that target patients with a specific, rather than general, combination of conditions, such as diabetes plus specific comorbidities [14]. Understanding the interaction between the care for diabetes and specific comorbidities might help in the development of tailored interventions.

A potentially valuable approach to integrating diabetes care with the care of other chronic conditions is to consider comorbidities as concordant or discordant with diabetes care [7]. In this general framework, conditions that share the same clinical management as diabetes are concordant with diabetes, and the presence of these conditions could cue providers to deliver the same or similar care as required for diabetes management, resulting in better diabetes care [7,15]. Discordant conditions do not share care with diabetes, and therefore would not cue providers to deliver recommended diabetes care, and may distract from diabetes care. Discordant conditions are those with treatments that either directly oppose diabetes care, such as requiring steroids that will increase blood sugar, or that simply do not share care with diabetes, such as using antacids for esophageal reflux. When a discordant condition is present, time limitations, competing demands, and other challenges may cause patients with diabetes to receive lower quality care for diabetes and the discordant condition as compared to having a single condition alone [8,15]. This framework could allow providers to target patients with multiple chronic conditions who are most at-risk for suboptimal diabetes care based on having fewer concordant conditions or more discordant conditions.

Previous investigations of the Piette and Kerr framework have been limited by the lack of a comprehensive list of diabetes concordant and discordant chronic conditions. Without a comprehensive set of concordant and discordant conditions, we cannot fully examine this framework for potential clinical use. Past studies have used a limited number of conditions categorized as concordant or discordant based on an overall impression of similar, or not, management, formed by context experts, researchers, and clinical practice [7,16]. Additionally, these categorizations have considered comorbidities as entirely concordant or discordant with diabetes [7,15,17]. However, diabetes care is complex and encompasses multiple testing and treatment goals [18]. Each comorbidity may be concordant or discordant with diabetes for one care goal and not for another. For instance, glaucoma may be concordant with the annual eye exam goal and discordant with the HbA1c testing goal. If it is found that concordant conditions are related to improved care in diabetes, and discordant conditions are related to worsened care, guidelines and interventions could highlight where synergistic care occurs and list conditions that might make the patients who have them benefit from extra attention to diabetes care. Also, public reporting could be stratified by concordant and discordant conditions to reflect differences in care and provide more personalized reports.

Our study aimed to provide more information for the future research and clinical use of the concordant-discordant framework by (1) increasing the number of conditions that can be characterized as concordant or discordant with diabetes, and (2) examining the number of care goals that are shared between concordant and discordant conditions. We aimed to provide researchers with a much needed tool to examine how comorbid chronic conditions might impact diabetes care.

## Methods

### Overview

We used Delphi methodology [16], a consensus-building technique that has been well-studied and is the basis for the RAND Appropriateness Method [19]. This technique is most effective when there is a lack of or inadequate information about an issue [16], such as exists in the literature defining chronic conditions as concordant or discordant with diabetes. Compared to committees and meetings, which can be dominated by a single individual, this technique considers all respondents’ opinions through anonymous reporting and feedback [16]. In our Delphi survey, we asked primary care providers (PCPs) to state whether each of 6 diabetes care goals were also care goals for a comprehensive set of outpatient-relevant chronic conditions. Conditions that shared the majority of care goals with diabetes were defined as concordant, and those that did not were defined as discordant. This study was approved by the University of Wisconsin Health Sciences Minimal Risk Institutional Review Board.

### Diabetes care goals

We chose 6 diabetes care goals that represent aspects of diabetes management across the pathophysiologic spectrum of diabetes that are necessary for most adult patients with diabetes (type 1 and type 2) and are related to short- and long-term health outcomes in diabetes [18]. The 6 diabetes care goals were: glycemic management, LDL cholesterol management, blood pressure management, kidney function monitoring, annual eye exam, and tobacco cessation counseling. These care goals can be measured in a standardized fashion across populations and health systems and are used for state and national diabetes performance metrics [18,20]. Despite some controversy in their use, there is general agreement that achieving these goals leads to better diabetes outcomes, and strong evidence shows that major complications are reduced if these goals are achieved [18,20].

### Chronic conditions

We built a list of 62 outpatient-relevant chronic condition categories (“conditions”) from a set of chronic condition categories previously used in multimorbidity research [21,22] and based on the AHRQ clinical classification system (CCS) of chronic medical conditions [23]. We further modified this set of condition categories to enhance the representation of cardiovascular, metabolic and mental health conditions by separating out conditions in these categories. Our 62 chronic condition categories encompass 1,412 ICD-9 codes. We counted a patient with multiple chronic conditions (multiple ICD-9 codes) within any single CCS category as having one chronic condition [21,22]. For example, a patient with the codes “malignant hypertension” (401.0) and “hypertensive heart disease” (402.0) is considered to have one condition, hypertension. Our modified chronic condition classification system is available online at no cost at www.hipxchange.org.

### Expert panel

Experts for our Delphi survey were recruited by an email sent to local PCPs who care for adult patients with chronic conditions. These PCPs (general internal medicine and family medicine physicians, physician assistants, and nurse practitioners) all practice at clinics affiliated with a large Midwestern academic medical center. We chose practicing PCPs as opposed to other specialists because they offer expertise in the management and care coordination of diabetes, along with a spectrum of multiple other chronic conditions.

The criterion for expert panel participation (practicing PCPs) was developed by all co-authors. Experts were chosen from the local academic health center for their academic clinical reputation and for convenience. The authors who conceptualized this study and designed the survey (E.M., R.G. and M.S.) did not serve as experts for the Delphi survey to avoid biasing results.

We initially contacted 161 academic PCPs by email for interest in participating in the Delphi survey. Sixteen PCPs agreed to participate and all 16 completed both rounds of the survey. The Delphi technique allows for selection of experts and does not require a representative sample of the population [24]. It also does not require a certain sample size, although 10–20 is typically considered sufficient [24]. Twelve PCPs were family medicine physicians, 2 were internal medicine physicians, 1 was an internal medicine physician assistant, and 1 was an internal medicine nurse practitioner. Most had more than 10 years of practice experience.

### Delphi survey procedure

We used a web-based survey to reduce undue peer influence on responses [16] (Additional file 1). The respondents remained anonymous to one another. The survey asked the PCPs to give their opinion on the management of chronic conditions in primary care. We asked providers if each of 6 listed care goals was “indicated” or “not indicated” in the management of each of the 62 listed chronic conditions. Management of a condition was explained as any testing or treatment that the provider would do beyond care for a normal, healthy individual. Providers were instructed to consider each patient as having only the listed chronic condition for each response. Diabetes was not mentioned anywhere on the survey to reduce the chance that respondents would think the hypothetical patient had both the listed chronic condition and diabetes and therefore respond that care was “indicated” for the chronic condition when they meant that it was indicated for the condition in the presence of diabetes. The survey had 372 condition-goal pairs (62 conditions for each of 6 care goals).

### Analysis

We determined diabetes concordance and discordance both on a goal-specific and summary condition-level for each chronic condition.

#### Goal-specific concordance and discordance

Goal-specific concordance of a chronic condition was defined as provider consensus opinion that a diabetes care goal was indicated for the chronic condition. If the care goal was not indicated, that condition had goal-specific discordance with diabetes. Conditions could be concordant for one diabetes care goal and discordant for another.

**Figure 1:**
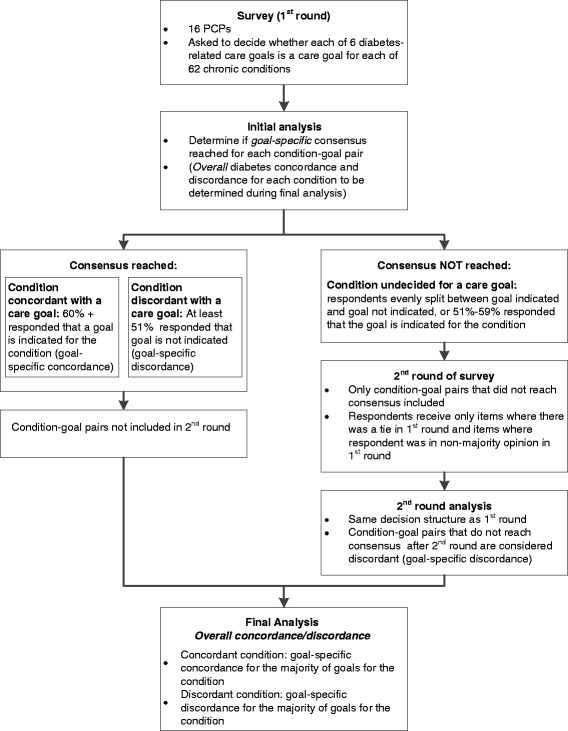
**Establishing chronic conditions’ goal-specific and overall concordance and discordance with diabetes.**

Provider consensus opinion for care goal concordance was reached when 60% of respondents agreed that a care goal was indicated for a chronic condition (60% majority opinion threshold). In the Delphi Method, the percentage agreement required to establish consensus is not definitive and typically ranges from 50-80% [25,26], where consensus levels higher than 80% are of unclear benefit [26]. We chose a 60% cut-off because at a higher threshold, over half of respondents would have to change their opinions in subsequent rounds on a specific care goal to move the majority opinion from concordant to discordant. As this is highly unlikely, we concluded that a 60% majority opinion accurately determines goal-specific concordance for a given condition-goal pair. Condition-goal pairs that were not considered concordant by 60% consensus opinion were considered discordant per the concordance and discordance framework*, i.e.,* any condition that is not concordant with diabetes care is considered discordant without needing a separate discordance threshold [7]. The 60% threshold was chosen prior to seeing the survey results (Figure 1.)

After the first round of surveying, condition-goal pairs that did not reach the 60% consensus threshold were re-addressed in a second survey round. We used only 2 rounds of the survey to determine consensus opinion as additional rounds have been shown not to improve outcomes [24]. The second round surveys were individualized, based on each respondent’s unique responses, to include only those condition-goal pairs for which the respondent was not in the majority opinion. The second round was conducted in waves, starting with those respondents who needed to be asked the fewest questions. As items reached consensus through the iterative process, they were dropped from further waves in round 2. This limited the time burden on participants and potential burn-out [24]. Condition-goal pairs that did not reach the 60% consensus threshold for concordance after the second round were defined as discordant.

#### Overall condition concordance and discordance analysis

We determined each chronic condition’s overall concordance or discordance by assessing whether a majority of care goals were concordant or discordant for each condition. Conditions that were concordant for the majority of care goals were established as having overall concordance with diabetes, and vice versa for discordant conditions.

## Results

After the first round of surveys, 339 of the 372 condition-goal pairs were categorized as concordant or discordant. Thirty-three condition-goal pairs did not reach consensus (19 condition-goal pairs were tied and 14 had a slight majority towards concordance) and went to the second round. After the second round of surveying, 9 condition-goal pairs of the 33 remained below the 60% concordance threshold and were declared discordant.

Unsurprisingly, the tobacco cessation counseling goal was unanimously indicated (concordant) for all conditions in the first round. As such, it could not be used to discriminate between conditions based on diabetes concordance, and was excluded from use in determining overall condition concordance. Therefore, overall condition concordance and discordance were established when 3 out of 5 goals were concordant or discordant, respectively.

Overall, 12 conditions were concordant with diabetes and 50 were discordant (see Table 1).The largest clinical group for concordant conditions was cardiovascular, whereas discordant conditions were distributed across multiple clinical groups. Discordant conditions included depression. Six conditions showed goal-specific concordance for all 5 goals except eye exam (Table 2). The other six concordant were concordant for 3 goals each. Thirty-one discordant conditions were discordant on all 5 goals, and 19 were discordant on all but 1 or 2 goals (Table 2).

**Table 1:** **Overall condition concordance and discordance**

**CONCORDANT CONDITIONS**	
**Cardiac, vascular, and pulmonary conditions**	**Genitourinary and reproductive conditions**
Acute myocardial infarction in past 2 years	Chronic renal failure
Cardiomyopathy and structural heart disease	Polycystic ovarian syndrome
Cerebrovascular disease	
Congestive heart failure	**Other conditions**
Coronary atherosclerosis	Obesity
Hyperlipidemia	
Hypertension	
Peripheral atherosclerosis	
Thrombosis and Embolism	
**DISCORDANT CONDITIONS**	
**Cardiac, vascular and pulmonary conditions**	**Mental Health Conditions**
Aneurysm	Anxiety disorders
Asthma or chronic obstructive pulmonary disease	Behavior disorders
Conduction disorder or cardiac dysrhythmia	Bipolar disorder
Congenital heart disease	Depression and depressive disorders
Heart valve disorder	Personality disorder
Non-thrombotic, non-athlerosclerotic vascular disease	Schizophrenia and psychotic disorders
Pulmonary heart disease	Sleep disorders
	Substance-use disorders
**Hematologic and Oncologic Conditions**	**Muscoskeletal conditions**
Anemia	Back problem
Malignant neoplasm	Gout or other crystal arthropathy
Sickle cell anemia	Osteoarthritis
**Gastrointestinal Conditions**	**Allergy and immunity conditions**
Chronic liver disease (excluding chronic hepatitis)	Allergic rhinitis
Chronic hepatitis	Immunity disorder
Chronic pancreatitis	Lupus
Diverticulosis/itis, intestinal malabsorption	Human immunodeficiency virus
Esophageal disorder	Rheumatoid arthritis
	Tuberculosis
**Neurologic Conditions**	**Genitourinary and Reproductive Conditions**
Dementia	Benign prostatic hypertrophy (BPH)
Migraines	Female infertility and GU anatomic disorders
Parkinson’s disease	Menopause and Perimenopause
Other central and peripheral nervous system disorders	Kidney and Vesicoureteral Disorders (excluding renal failure)
Multiple sclerosis	
Paralysis	**Other conditions**
Epilepsy	Amyloidosis
	Chronic skin ulcer
**Endocrine conditions**	Cystic fibrosis
Thyroid Disorder	Degenerative eye problem
	Non-cardiac congenital disorder
	Sarcoidosis

**Table 2:** **Goal-specific concordant and discordant conditions**

**CONCORDANT CONDITIONS**	
**Concordant on all goals** ***except eye exam***	**Concordant on all** ***goals except annual eye exam and kidney function monitoring***
Acute myocardial infarction in past 2 years	Hyperlipidemia
Coronary atherosclerosis	Polycystic ovarian syndrome
Peripheral atherosclerosis	Obesity
Hypertension	
Cerebrovascular disease	**Concordant on all goals** ***except annual eye exam and blood sugar management***
Chronic renal failure	Congestive heart failure
	Cardiomyopathy and structural heart disease
	Thrombosis and embolism
**DISCORDANT CONDITIONS**	
**Discordant on all goals**	**Discordant on all goals** ***except kidney function monitoring***
Asthma or COPD	Gout or other crystal arthropathy
Chronic hepatitis	Sickle cell anemia
Diverticulosis/itis, intestinal malabsorption	Rheumatoid arthritis
Esophageal disorder	
Chronic pancreatitis	**Discordant on all goals** ***except annual eye exam***
Female infertility/GU anatomic disorders	Degenerative eye problem
Benign prostatic hypertrophy (BPH)	
Epilepsy	**Discordant on all goals** ***except blood pressure management***
Multiple sclerosis	Conduction disorder or cardiac dysrhythmia
Parkinson’s disease	Congenital heart disease
Back problem	Chronic liver disease (excluding chronic hepatitis)
Osteoarthritis	Menopause and perimenopause
Anemia	Paralysis
Malignant neoplasm	Migraines
Allergic rhinitis	Dementia
Immunity disorder	Other central and peripheral nervous system disorders
Tuberculosis	Sleep disorders
Human immunodeficiency virus	
Anxiety disorders	**Discordant on all goals** ***except blood pressure management and kidney function monitoring***
Depression	Kidney and vesicoureteral disorders (excluding renal failure)
Bipolar disorder	Lupus
Substance-use disorder	
Personality & psychogenic disorders	**Discordant on all goals** ***except blood pressure management and cholesterol management***
Schizophrenia and psychotic disorders (excluding mood disorders)	Heart valve disorder
Behavioral disorders	Aneurysm
Chronic skin ulcer	Non-thrombotic, non-athlerosclerotic vascular disease
Thyroid disorder	Pulmonary heart disease
Amyloidosis	
Sarcoidosis	
Cystic Fibrosis	
Non-cardiac congenital anomaly	

## Discussion

Our study determined the goal-specific and overall condition concordance and discordance with diabetes of a comprehensive set of chronic conditions using primary care provider expert opinion. Among 62 ambulatory care-relevant chronic conditions, 12 conditions were concordant with diabetes care, and were all diabetes risk factors or complications. The remaining 50 conditions (including depression) were discordant, showing limited overlap with diabetes care goals. Our results show that not all conditions are equal in how they interact with diabetes care, and conditions can be categorized as concordant or discordant overall and specific to diabetes care goals. We also found that not all PCPs’ opinions matched current clinical practice guidelines. This work has implications for future research to improve clinical guidelines, public reports and interventions for patients with diabetes and multiple chronic conditions.

Previous studies on the diabetes concordance-discordance framework have used the study authors’ opinions [27-31] or the nominal group technique [15] to determine concordance and discordance. In contrast, the Delphi Method mitigates undue influence from other members of the group, as respondents do not know the individual responses of other group members. Our approach employed the professional judgment of PCPs who are experts at managing and coordinating the care of multimorbid patients. As the front-line providers for these complex patients, their opinions on care are most relevant for study, resulting in findings that are highly applicable to clinical practice. A survey of PCPs shows the complex cognitive and clinical realities of caring for multimorbid patients with diabetes [32]. To date, no study has analyzed as large or comprehensive a set of diabetes care goals and chronic conditions [15,27,29-31]. We added to the number of conditions categorized as concordant and discordant, and validated concordance and discordance categorizations done in previous work (Table 3). We compared the overall condition concordance-discordance results to previous literature on the subject [33]. Our results are generally consistent with the limited number of conditions categorized in prior literature.

**Table 3:** **Comparison of our findings to previous work**

**Concordant conditions**		**Discordant conditions**	
**Confirmed concordant**	**Newly found to be concordant**	**Confirmed discordant**	**Newly found to be discordant**
acute myocardial infarction	cardiomyopathy	osteoarthritis	heart valve disorder
congestive heart failure	thrombosis and embolism	back problem/pain	aneurysm
coronary atherosclerosis	obesity	mental illness	non-thrombotic, non-atherosclerotic vascular disease
peripheral atherosclerosis	polycystic ovarian syndrome	GERD	benign prostatic hypertrophy
hypertension		irritable bowel syndrome	female infertility and genitourinary anatomic disorders
cerebrovascular disease		hepatitis	sickle cell anemia
chronic renal failure		chronic obstructive	immunity disorder
		pulmonary heart disease	tuberculosis
		multiple sclerosis	thyroid disorder
			amyloidosis
			sarcoidosis
			cystic fibrosis

Clinically, the conditions we found to be concordant comprise vascular, metabolic and renal conditions that share pathophysiology with diabetes and co-occur frequently. We expected these conditions to be concordant based on the conceptual model and previous work. All concordant conditions have goal-specific concordance with diabetes for LDL and blood pressure management, highlighting the importance of cardiovascular risk management for both diabetes and to provide synergistic benefit to common comorbidities.

Most conditions were discordant, even those whose care is considered of special importance in diabetes. For example, depression is a concerning comorbidity in diabetes, as it is associated with worse outcomes and there is a suggestion of a bidirectional relationship for poor control between diabetes and depression [34]. Our study found that depression was viewed as discordant with diabetes, sharing none of the assessed care goals. This suggests that, for a patient with diabetes and depression, diabetes care goals would be addressed without additional cuing from depression (no overlap in care), and that depression care goals could increase the health care workload and lead to missed care. Alternatively, despite the categorization of depression as discordant, patients with depression and diabetes might receive extra attention on diabetes care, as it is fairly well known that depression is associated with worse diabetes outcomes, and providers might put extra emphasis on treating these patients. This was not assessed in our study and should be studied in future work.

Our study also expands the concordance-discordance framework from solely overall condition concordance-discordance to include concordance-discordance of conditions with specific diabetes care goals. Goal-specific concordance and discordance adds potentially valuable clinical detail for use with the concordance-discordance framework. This expanded knowledge can be used in future research to understand the interaction of conditions in more detail than can be done with only overall condition concordance and discordance [8]. Of the 50 discordant conditions, 19 were concordant with diabetes on 1–2 individual diabetes care goals. Although discordant overall, these conditions still have the potential to interact synergistically with diabetes for those care goals with which they are concordant. These conditions might therefore improve diabetes care for these goals while distracting from diabetes care for other goals. Overall condition concordance and discordance remains important to demonstrate and summarize which conditions, as a whole, share care (or not) with diabetes and might be more likely to interact favorably with diabetes care (or not).

Interestingly, there was some discrepancy between PCP perceptions and guideline recommended care, which may reflect lower familiarity with less commonly seen conditions. The discrepancy also highlights an opportunity to correct cognitive models for concordant conditions to improve clinical care for patients whose comorbidities may confer additive risk. For example, PCPs perceived rheumatoid arthritis as discordant with diabetes on the goal level with glycemic, blood pressure, and lipid management. However, rheumatoid arthritis, like diabetes, increases cardiovascular disease risk, and requires attention to blood pressure, lipid control, and glycemic control [35,36]. Our findings align with results of prior work by our group showing that Medicare patients with RA and diabetes actually received fewer A1c tests than diabetes patients without rheumatoid arthritis [37], fitting with provider perceived discordance despite shared physiologic cardiovascular risk.

### Clinical implications

The major goal of this study was to provide more information for the future research and subsequent clinical use of the concordant-discordant framework by (1) increasing the number of conditions that can be characterized as concordant or discordant with diabetes, and (2) examining the number of care goals that are shared between concordant and discordant conditions [7]. If future research demonstrates differences in clinical outcomes associated with concordant and discordant conditions, then there are several potential clinical implications for guidelines, interventions and public reporting of this more detailed and thorough application of the concordance-discordance framework for patients with diabetes and multiple chronic conditions. Current guidelines, for the most part, are disease-centric. While recent diabetes guidelines suggest individualized targets for patients with specific chronic conditions, the guidelines still have diabetes as the central condition and discuss diabetes care goals, without incorporating the non-diabetes care goals of most comorbidities, especially discordant comorbidities. One approach to guideline use in multimorbidity is to electronically cross-reference guidelines and create a patient-specific guideline listing all the patient’s conditions and the conditions’ associated care goals [9]. Using goal-specific concordance, this list could be integrated across conditions to list all recommended care goals for that patient, for all conditions, and the conditions the care goals benefit (for example, blood pressure control benefits diabetes and heart failure but not necessarily depression). This approach would also help patients and providers discuss risks and benefits of each care goal across all conditions to see which goal would give the largest health benefit and/or best fits their personal care priorities [9,10]. Interventions to improve care can apply integrated care goal and risk-benefit discussions across all conditions. These interventions can target patients most at risk for suboptimal care based on their concordance and discordant conditions, if future work shows that patients with certain multimorbidity profiles are more at risk than others. Finally, public reports of care quality could be stratified by comorbidity type so that patients are compared to patients more similar to them, giving more meaningful reports. These reports could consider using non-disease specific outcomes, such as reporting on care goals that overlap between conditions, care goals that give the greatest benefit with least risk across conditions, or functional outcomes [13].

### Limitations

A potential limitation of our study is that our expert panel included a variety of PCPs (e.g., family medicine, internal medicine). While it is possible that the different specialties of the providers could bias our results, we think it is more likely that the diversity of our panelists best represents the range of PCPs and their opinions. Additionally, it is possible that the PCPs who chose to participate in the panel have a special interest in research or quality improvement. If this were the case, we would expect the result to bias towards more conditions having care goals indicated and more concordance. We also chose certain diabetes care goals based on current guidelines and publicly reported quality metrics, but there are other care goals that are relevant to diabetes. We didn’t include in our survey additional diabetes care goals that are used in quality reporting or recommended in diabetes guidelines, including foot care and lifestyle counseling, as achievement of these goals is harder to consistently quantify between providers and health systems. As concordance and discordance in the original framework is defined as sharing management and pathophysiology, or not, this model is inherently provider-centric, based on care processes and treatments that are ordered by the provider and assessed in the clinic. Future work could assess the patient’s perspective on concordance and discordance by assessing patient opinion on overlap in self-care, such as exercise and diet changes. We recognize that more factors than care goal overlap between chronic conditions have an influence care quality and should be considered in the development of future guidelines and interventions. Patient contextual elements must be included such as age and socioeconomic status [38], since older adults require different care than younger adults [10] and lower socioeconomic status is a risk factor for an earlier age of onset for chronic conditions [4]. Finally, one goal of our study was to determine care goal overlap. Future work should determine the clinical impact of this overlap, and if concordance and discordance impact care quality differently, especially for different care goals (e.g., HbA1c testing vs. HbA1c control, HbA1c control versus BP control).

## Conclusions

Our study shows that PCPs perceive the care of diabetes to overlap with the care of several other chronic conditions, especially chronic conditions that are risk factors for or complications of diabetes. Other chronic conditions, including depression, an important diabetes comorbidity, were not perceived as having overlapping care with diabetes. As our approach differentiates overall condition concordance-discordance and goal-specific concordance-discordance, our results highlight potentially helpful overlaps in care that are not evident with overall condition discordance. This knowledge will be especially useful as we move towards guidelines and interventions that focus on condition interactions in multiple chronic conditions, rather than considering each condition in isolation. Understanding the interactions between conditions at the level of care goals could be a key to identifying patients with diabetes most at-risk for suboptimal care due to their other chronic conditions, and to devising system-level interventions to target and improve care for these patients.

## Additional file

Addditional file 1:
**Screenshot of one page of the we -based Delphi survey.**

## Abbreviations

PCP: Primary Care Provider
CCS: Clinical Classification System
