# Supplementary material for: Establishing chronic condition concordance and discordance with diabetes: a Delphi study
Source: BMC Fam Pract. 2015 Mar 28;16:42. doi: 10.1186/s12875-015-0253-6 (PMC4391600; doi:10.1186/s12875-015-0253-6)
Supplement: Addditional file 1: — Screenshot of one page of the we -based Delphi survey. [file 12875_2015_253_MOESM1_ESM.pdf]

ADDITIONAL FILE 1: DELPHI SURVEY SAMPLE

Please identify if, in your practice, the following care is indicated for a typical adult patient who has each specific chronic condition. Assume that the patient has only this chronic condition. Please click only where you would typically undertake the proposed care management strategy.

Cardiac, vascular and pulmonary conditions

	Tobacco cessation counseling: Click if indicated	Blood pressure management: Click if indicated	LDL cholesterol management: Click if indicated	Kidney function monitoring: Click if indicated	Annual eye exam: Click if indicated	Blood sugar management: Click if indicated
Acute myocardial infarction in past 2 years	☐	☐	☐	☐	☐	☐
Congestive heart failure	☐	☐	☐	☐	☐	☐
Coronary atherosclerosis	☐	☐	☐	☐	☐	☐
Peripheral atherosclerosis	☐	☐	☐	☐	☐	☐
Hyperlipidemia	☐	☐	☐	☐	☐	☐
Hypertension	☐	☐	☐	☐	☐	☐
Conduction disorder or cardiac dysrhythmia	☐	☐	☐	☐	☐	☐
Heart valve disorder	☐	☐	☐	☐	☐	☐
Congenital heart disease	☐	☐	☐	☐	☐	☐
Cerebrovascular disease	☐	☐	☐	☐	☐	☐
Aneurysm	☐	☐	☐	☐	☐	☐
Cardiomyopathy and structual heart disease	☐	☐	☐	☐	☐	☐
Non-thrombotic, non-athlerosclerotic vascular disease	☐	☐	☐	☐	☐	☐
Pulmonary heart disease	☐	☐	☐	☐	☐	☐
Asthma or chronic obstructive pulmonary disease	☐	☐	☐	☐	☐	☐
Thrombosis and embolism	☐	☐	☐	☐	☐	☐
